# Growth of breast cancer recurrences assessed by consecutive MRI

**DOI:** 10.1186/1471-2407-11-155

**Published:** 2011-04-28

**Authors:** Ingrid Millet, Emmanuelle Bouic-Pages, Denis Hoa, David Azria, Patrice Taourel

**Affiliations:** 1 Centre Hospitalier Universitaire Lapeyronie, Montpellier, France; 2 CRLC Val d'Aurelle, Montpellier, France

## Abstract

**Background:**

Women with a personal history of breast cancer have a high risk of developing an ipsi- or contralateral recurrence. We aimed to compare the growth rate of primary breast cancer and recurrences in women who had undergone prior breast magnetic resonance imaging (MRI).

**Methods:**

Three hundred and sixty-two women were diagnosed with breast cancer and had undergone breast MRI at the time of diagnosis in our institution (2005 - 2009). Among them, 37 had at least one prior breast MRI with the lesion being visible but not diagnosed as cancer. A linear regression of tumour volume measured on MRI scans and time data was performed using a generalized logistic model to calculate growth rates. The primary objective was to compare the tumour growth rate of patients with either primary breast cancer (no history of breast cancer) or ipsi- or contralateral recurrences of breast cancer.

**Results:**

Twenty women had no history of breast cancer and 17 patients were diagnosed as recurrences (7 and 10 were ipsi- and contralateral, respectively). The tumour growth rate was higher in contralateral recurrences than in ipsilateral recurrences (growth rate [10^-3 ^days^-1^] 3.56 vs 1.38, p < .001) or primary cancer (3.56 vs 2.09, p = 0.01). Differences in tumour growth were not significant for other patient-, tumour- or treatment-related characteristics.

**Conclusions:**

These findings suggest that contralateral breast cancer presents accelerated growth compared to ipsilateral recurrences or primary breast events.

## Background

Better knowledge of breast cancer growth rates has many implications for diagnosis, treatment and follow-up. Recent preclinical studies suggest that cancer treatments such as radiotherapy, chemotherapy, and surgery can induce accelerated repopulation and more aggressive disease arising from surviving tumour cells [1-3]. Among aggressiveness characteristics, the tumour growth rate is important but seldom available as breast cancers are not followed-up without treatment. A few observational studies have retrospectively analyzed missed cancers on mammograms to evaluate breast cancer growth rates. However, mammography is neither the most sensitive nor the most reliable imaging modality for detecting or measuring breast cancer.

Breast magnetic resonance imaging (MRI) has been shown to be more accurate than ultrasound and mammography in estimating the local extent of breast cancer and assessment of tumour size [4,5]. Moreover, as MRI is more sensitive than mammography and ultrasound for breast cancer diagnosis, it is used for screening women at high risk of breast cancer, follow-up of breast cancer survivors, and other situations, including "problem-solving" when standard clinical and imaging evaluation do not provide a clear diagnosis [6-9].

Nevertheless, high specificity is lacking, and some cancers, although visible as enhancing lesions, are not initially diagnosed and undergo several follow-up MRI examinations, thus enabling tumour growth assessment [10].

In this context of breast cancers that may have undergone serial MRI examinations before diagnosis, we hypothesized that if previously treated primary breast cancer can promote aggressive recurrences in the clinical setting, then we could observe an increased growth rate of such recurrences. We conducted a retrospective study and assessed growth rates of 20 primary breast cancers, 10 ipsilateral recurrence and 7 contralateral recurrences of breast cancers in all women that were diagnosed with breast cancer and had undergone several breast MRI scans with a visible lesion prior to diagnosis.

## Methods

Approval for this retrospective study was obtained from our institutional review board, which waived the requirement for informed consent. This study was compliant with the Health Insurance Portability and Accountability Act. The study is reported according to the STROBE statement.

### Participants

**Figure 1:**
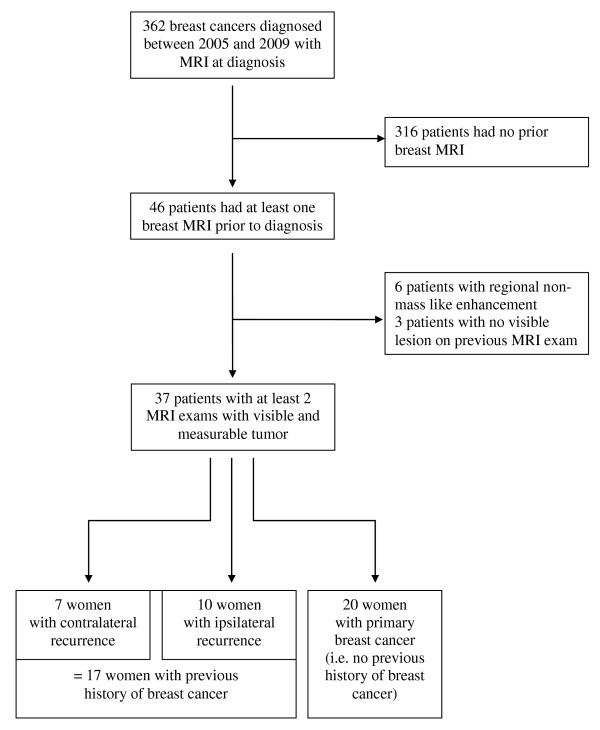
**Flow diagram**.

This retrospective study examined original histological reports of 362 consecutive patients with breast cancer diagnosed by biopsy in our institution and with breast MRI performed at time of diagnosis, between 1 January 2005 and 31 December 2009. 46 patients had undergone at least one other breast MRI scan prior to diagnosis. Three patients with cancer not visible on previous MRI scans (no enhancing lesion) were excluded, along with 6 patients with regional non-mass like enhancement (lesion volume not measurable with reliability). After exclusions, there were 37 cases for analysis - 7 women with ipsilateral breast cancer recurrence, 10 women with contralateral breast cancer recurrence and 20 women with primary breast cancer (Figure 1).

Multiple MRI examinations may have been performed prior to cancer diagnosis because: (a) in 24 cases the lesion was considered as BIRADS 3 with systematic follow-up planned; (b) in 7 cases the patient underwent MRI-guided or US-guided biopsy with a non-malignant result, the lesion was thus monitored by MRI; or (c) in 6 cases there was failure to initially detect a small breast lesion due to diffuse breast enhancement.

Documented information included patient age at diagnosis, patient history of breast cancer and treatment of primary cancer, menopausal status, breast tumour size, type, grade, mitotic count.

### MRI technique and image analysis

All participants underwent dynamic, contrast-enhanced breast MRI. Minimum standard criteria were required for each MRI study performed: a 1.5-T magnet, a dedicated breast-surface coil, and one image obtained before and dynamic images obtained after the administration of contrast material, with three-dimensional, T1-weighted, gradient-echo sequences. Spatial-resolution criteria included voxels smaller than 0.7 mm in the frequency-encoding direction, smaller than 1 mm in the phase-encoding direction, and 3 mm or smaller in the slice direction, thus providing full coverage of the breast.

Two readers, who were blinded to the pathological tumour size, independently reviewed the randomized breast MRI. To avoid confusion with other breast lesions, the readers were made aware of the location of the lesions of interest. For each lesion, the greatest axial diameters, greatest perpendicular diameters, and number of sections were measured and recorded.

### Growth model

The tumour volume was estimated using the formula for obloid spheroids

with a, b, c denoting the mean of the two readers' diameters measurements.

Several studies showed that decelerating equations provided better fits for the growth of human breast cancer tumours than the exponential law. Such growth curves can be described by a logistic function or a Gompertz function [11-14]. Both Spratt and Weedon-Fekjaer used a variant of the log-normal logistic growth model with a maximum tumour volume of 40 cell doublings, equalling a ball of 128 mm in diameter, after testing several models on a clinical dataset that mostly consisted of overlooked tumours at earlier mammograms. In the present study, we used the same variant of the general logistic growth model which implies almost exponentional growth for the smallest tumours with growth decelerating as the tumours approach their supposed maximum volume [11,12,15].

Mathematically, this equation gives the following specification of tumour volume, V(t), as a function of time, t:

where b is the growth rate, V_max _is the maximum tumour volume (set for a tumour of 128 mm in diameter), and V_cell _is the volume of one cell. The following linear form of this equation is obtained by solving for b:

Therefore, b can be determined by a linear regression of the time and volume obtained from MRI data (as all calculations in the present paper use a relative cancer time, the choice of V_cell _does not affect the given estimates).

Actual tumour doubling time DT_act _was calculated for each patient based on the first recorded tumour volume V using the following equation:

### Endpoint and objectives

The primary endpoint was the tumour growth rate. Secondary endpoints were tumour volumes and tumour sizes.

The primary objective of the present study was to compare tumour growth rates between women without personal history of breast cancer, with ipsilateral recurrence, and with contralateral recurrence. Secondary objectives were to identify whether patient-, tumour- or treatment-related parameters were linked to tumour growth rates and to assess the reproducibility and accuracy of tumour volume measurements.

### Statistical methods

The three female groups were compared for patient and disease characteristics in order to verify group comparability and detect any confounding factor using the Kruskal-Wallis one-way analysis of variance in case of continuous variables and of the χ2 test or Fisher's exact test in case of categorical variables.

To test for differences in the distribution of tumour growth rates across groups, menopausal status and histological grades, we used the Kruskal-Wallis one-way analysis of variance.

The Bonferroni correction was used to assess significance in comparisons of growth rates among subgroups.

Tumour characteristics recorded as continuous variables (volume, hormone receptors, Ki67, mitotic count) were dichotomized using the median as cut-off point.

Comparison of the distribution of tumour growth rates between histological subtypes and dichotomized tumour characteristics were performed by the Wilcoxon test.

Interobserver agreement in the volume measurements and agreement between the major diameter measured on MRI and at pathologic examination was assessed by computing intraclass correlation coefficients.

P < .05 denoted statistical significance.

Computations were carried out using SAS version 9.2 software.

This study was not registered.

### Role of the funding source

There was no funding source for this study.

## Results

### Participants

Table 1 details the patient characteristics.

**Table 1:** Characteristics of patient groups

	Primary breast cancer	Recurrence of breast cancer	Ipsilateral recurrence	Contralateral recurrence	P
Characteristic	(n = 20)	(n = 17)	(n = 7)	(n = 10)	
**Age at detection (years)**					
Median (range)	56 (40-72)	60 (44-78)	57 (53-64)	62 (44-78)	0.61
**Menopausal status**					
Pre-	6 (30%)	1 (6%)	1 (14%)	0 (0%)	
Post-, with HT	3 (15%)	0 (0%)	0 (0%)	0 (0%)	0.11
Post-, without HT	11 (55%)	16 (94%)	6 (86%)	10 (100%)	
**Mutation**					
BRCA 1	2 (10%)	0 (0%)	0 (0%)	0 (0%)	0.24
BRCA 2	0 (0%)	1 (5.9%)	1 (14%)	0 (0%)	
**Delay between cancers**					
		10 (2 - 19)	13 (9-19)	8 (2-17)	.003
**Histologic type of primary cancer**					
IDC/ILC		12 (75%)	6/7 (86%)	6/9 (67%)	
DCIS		4 (25%)	1/7 (14%)	3/9 (33%)	
**Treatment of primary breast cancer**					
Surgery					0.48
Breast-conserving surgery		15 (88%)	7/7 (100%)	8/10 (80%)	
Mastectomy		2 (12%)	0/7 (0%)	2/10 (20%)	
Radiotherapy		16 (94%)	7/7 (100%)	9/10 (90%)	1
Chemotherapy		8 (47%)	2/7 (29%)	6/10 (60%)	0.33
Hormonal therapy		4 (27%)	1/6 (17%)	3/9 (33%)	0.60
**Location of cancer**					
Inner	3 (15%)	5 (29%)	1 (14%)	4 (40%)	
Central	4 (20%)	1 (6%)	1 (14%)	0 (0%)	0.43
Outer	13 (65%)	11 (65%)	5 (17%)	6 (60%)	
**Histology of new cancer**					1.00
Invasive carcinoma	18 (90%)	15 (88%)	6 (86%)	9 (90%)	
SBR 1	9 (50%)	4 (22%)	2 (33%)	2 (22%)	
SBR 2	5 (28%)	9 (60%)	3 (50%)	6 (67%)	0.51
SBR 3	4 (22%)	2 (13%)	1 (7%)	1 (11%)	
DCIS	2 (10%)	2 (12%)	1 (14%)	1 (10%)	
**Tumour size**					
Maximum tumour diameter at pathologic review (mm)	9 (0 - 28)	10 (2 - 35)	10 (2 - 18)	8.5 (5 - 35)	0.97
Initial volume (mm3)	244 (14 - 4837)	121 (8 - 2806)	121 (26 - 612)	116 (8 - 2806)	0.44
Final volume (mm3)	357 (36 - 4373)	425 (68 - 4319)	359 (100 - 1355)	458 (68 - 4319)	0.90
**Hormone receptors**					
Oestrogen receptors	80 (0-100)	95 (20 - 100)	90 (60 - 100)	100 (20 - 100)	0.06
Progesterone receptors	30 (0 - 95)	40 (0 - 95)	25 (2 - 95)	55 (0 - 90)	0.99
**Ki67**					
Median (range)	6 (1 - 35)	12 (2 - 40)	12 (5 - 30)	12.5 (2 - 40)	0.09
**Mitotic count**					
Median (range)	2 (0 - 31)	7 (1 - 20)	3.5 (1 - 10)	9 (1 - 20)	0.20

All participants were over 40 years old (median age: 56 y., range: 40 - 72 years).

There was a higher rate of post-menopausal women in the recurrence group and none of them had hormone therapy (p = 0.02). Seven patients (36%) were pre-menopausal and three women (15%) received hormone therapy. Only 3 women were BRCA carriers, 2 with BRCA 1 mutation in the primary cancer group, and 1 with BRCA 2 mutation with an ipsilateral recurrence.

Patients with no personal history of breast cancer and patients with ipsi- or contralateral recurrences did not differ in terms of age and mutation status (Table 1).

All recurrences were late recurrences, which were new primary tumours, diagnosed at least 2 years after the initial diagnosis of primary cancer, with a median time interval of 10 years (range 2 - 19 years). All patients with a personal history of breast cancer received radiation therapy, except for one woman who underwent mastectomy. Contralateral recurrences were diagnosed earlier than ipsilateral recurrences (median: 8 years after primary cancer vs 13 years, p = 0.003).

The median time interval between initial and final MRI was 659 days (range, 134 - 1290 days).

### Tumours

There were 32 invasive ductal carcinomas, 1 invasive lobular carcinoma, and 4 ductal carcinomas in situ. The median maximum tumour diameter at pathologic review was 10 mm (range 0 - 35 mm). One tumour was entirely removed at biopsy and no macroscopic cancer was measurable at postsurgical pathology.

Initial and final tumour volumes on the first and last MRI scans were, respectively, 139 mm^3 ^(8 - 4837) and 425 mm^3 ^(36 - 4373), with no significant difference between the primary cancer and recurrence groups.

Recurrences and primary cancers did not differ in terms of histological type, size, location, grade, hormone receptors, Ki67 level, or mitotic count (Table 1).

### Growth rate

Table 2 shows a comparison of growth rates in terms of patient-, tumour- or treatment-related characteristics.

**Table 2:** Comparison of tumour growth rates

Characteristic	n	Growth rate b (10^-3 ^days^-1^)	Actual doubling time DT_act _(days)	P
**Age at detection**				
< 59 y. (median)	18	2.01 (-1.41 - 5.77)	341 (-685 - 785)	0.79
>= 59 y.	19	2.21 (-0.12 - 5.79)	343 (-6797 - 4470)	
**Menopausal status**				
Pre-	7	2.73 (1.25 - 3.98)	293 (193 - 651)	
Post-, with hormone therapy	3	-0.12 (-1.41 - 2.18)	-685 (-6797 - 343)	0.14
Post-, without HT	27	2.22 (0.18 - 5.79)	344 (129 - 4470)	
**Personal history**				
No previous breast cancer	20	2.09 (-1.41 - 3.98)	284 (-6797 - 4470)	
Ipsilateral recurrence	7	1.38 (1.10 - 2.22)	581 (344 - 710)	0.004
Contralateral recurrence	10	3.56 (1.65 - 5.79)	221 (129 - 481)	
**Contralateral recurrence**				
No	27	1.69 (-1.41 - 3.98)	390 (-6797 - 4470)	0.001
Yes	10	3.56 (1.65 - 5.79)	221 (129 - 481)	
**Treatment of primary breast cancer**				
**Surgery**				
Breast-conserving surgery	15	2.12 (1.10 - 5.77)	355 (129 - 710)	0.19
Mastectomy	2	4.02 (2.26 - 5.79)	237 (130 - 344)	
**Radiotherapy**				
No	1	5.79 (5.79 - 5.79)	130 (130 - 130)	0.10
Yes	16	2.17 (1.10 - 5.77)	349 (129 - 710)	
**Chemotherapy**				
No	9	2.02 (1.10 - 5.79)	390 (129 - 710)	0.71
Yes	8	2.24 (1.19 - 5.76)	344 (141 - 660)	
**Hormonal therapy**				
No	11	2.26 (1.10 - 5.79)	344 (129 - 710)	0.90
Yes	4	2.51 (1.19 - 5.76)	335 (141 - 660)	
**Location of cancer**				
Inner	8	2.09 (1.10 - 5.79)	376 (129 - 785)	
Central	5	2.89 (1.38 - 3.33)	293 (245 - 604)	0.70
Outer	24	2.17 (-1.41 - 5.76)	330 (-6797 - 4470)	
**Histology**				
Invasive carcinoma	33	2.21 (-0.12 - 5.79)	343 (-6797 - 4470)	0.17
In situ carcinoma	4	0.75 (-1.41 - 5.76)	401 (-685 - 2643)	
**Grade**				
SBR 1	13	2.00 (0.18 - 3.82)	409 (192 - 4470)	
SBR 2	14	2.59 (-0.12 - 5.79)	273 (-6797 - 710)	0.17
SBR 3	6	2.81 (1.10 - 3.35)	284 (234 - 785)	
**Tumour initial volume**				
< 139 mm3 (median)	18	2.73 (0.91 - 5.79)	274 (129 - 817)	0.04
≥ 139 mm3	19	1.52 (-1.41 - 5.76)	399 (-6797 - 4470)	
**Oestrogen receptors**				
< 90% (median)	15	2.22 (-0.12 - 5.77)	293 (-6797 - 4470)	0.75
≥ 90%	17	2.12 (0.91 - 5.79)	355 (130 - 817)	
**Progesterone receptors**				
< 35% (median)	16	1.95 (-0.12 - 3.84)	318 (-6797 - 785)	0.44
>= 35%	16	2.22 (0.18 - 5.79)	343 (129 - 4470)	
**Ki67**				
< 7.5% (median)	13	2.18 (-0.12 - 5.77)	265 (-6797 - 4470)	0.67
≥ 7.5%	13	2.26 (1.10 - 5.79)	344 (130 - 710)	
**Mitotic count**				
< 3.5 (median)	16	2.01 (0.18 - 3.98)	399 (192 - 4470)	0.17
≥ 3.5	16	2.81 (-0.12 - 5.79)	284 (-6797 - 785)	

The overall growth rate of tumours was 2.18 10^-3 ^days^-1 ^(range: -1.41 - 5.79, actual doubling time: median 343 days, range -6797 - 4470). Two tumours demonstrated a decrease in volume between the initial and final MRI scans.

**Figure 2:**
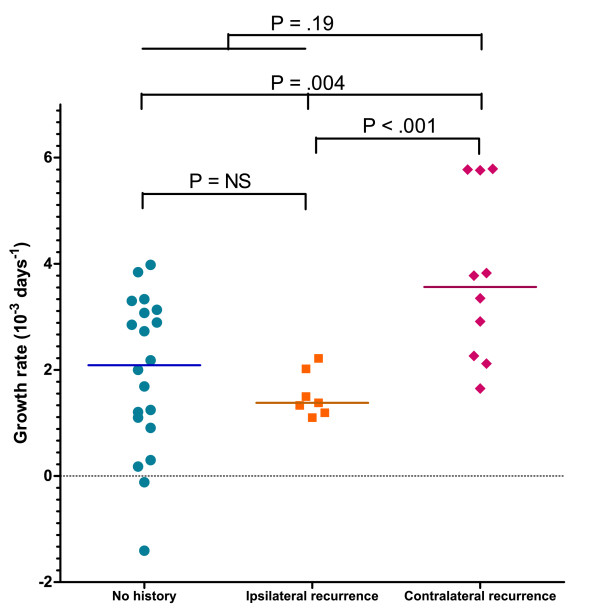
**Comparison of tumour growth rates between patients with primary breast cancer, ipsilateral, or controlateral recurrence of breast cancer**.

Analysis showed a significant accelerated growth rate for contralateral recurrences compared to ipsilateral recurrences or primary cancers (growth rate [10^-3 ^days^-1^]: 3.56 vs 1.38 vs 2.09, respectively, p = 0.004) (Figure 2).

No significant differences in growth rate were found between primary cancers and ipsilateral recurrences (2.09 vs 1.38, p = NS).

When dichotomizing the tumours according to the median initial volume (139 mm^3^), the largest tumours showed lower growth rates than the smallest tumours (1.52 vs 2.73, p = 0.04), which is consistent with the hypothesis of a decelerating tumour growth model.

The growth rate ranges of breast tumours of different types and grades were wide and overlapped substantially: growth rates did not differ in terms of other patient characteristics (age, mutation status, menopausal status), tumour characteristics (histological type, grade, hormone receptors, Ki67 level, mitotic count), or previous cancer therapy.

### Study of agreement

Inter-reader reliability for volume measurements and agreement for maximum diameter measurement between MRI and pathological examination assessed by the intraclass correlation coefficient were respectively 0.96 (95% CI: 0.94 - 0.97) and 0.91 (95% CI: 0.83 - 0.95).

## Discussion

Although preclinical studies suggest cancer treatments can accelerate tumour growth, assessing tumour progression prospectively in patients without therapeutic intervention is not feasible due to obvious ethical issues.

To date and to our knowledge, no observational study has investigated the growth rate of late breast cancer recurrences with MRI. Very few simulation studies and retrospective clinical analyses have been published on the growth rate of human breast cancers [16]. Moreover, these studies were often based on mammography, which has a lower sensitivity and ability to evaluate tumour size as compared to MRI [12,13,15]. Growth rate estimations from screening populations also have weak points as chosen mathematical models of tumour growth and screening test sensitivity are controversial [13,15]. One author even tried to evaluate the tumour growth rate with the lesion not always being visible in the imaging examinations, while setting a default size for non-visible tumours [16,17].

Since MRI allows for accurate measurement of tumour volume, and as several MRI exams are sometimes needed to suggest malignancy, breast tumour growth rates could be measured in a retrospective fashion. It was therefore the first goal of the present study to assess whether breast cancer recurrences have an accelerated growth rate compared to primary cancers.

The median growth rate of the breast cancers in our study (DTact: 343 days, range -6797 - 4470), which was measured volumetrically using serial MRI, was lower than previously reported measured or estimated growth rates [12,15,17]. This difference might be explained by the wide growth rate range and potential selection bias due to overlooked MRI lesions being small cancers [12,15,16].

The main weaknesses of our study are a small sample size and a mixed population. Conclusions are drawn from 37 patients with both ductal and lobular carcinomas. Despite the small sample size, univariate analysis suggests that contralateral breast cancer recurrences may show accelerated tumour growth compared to ipsilateral recurrences and primary cancers, with statistical significance. Unfortunately, multivariate analysis of our data is limited by the sample size.

Mathematical modelling of human breast cancer growth is still controversial, and differences in growth rates between the largest and smallest tumours in our study suggest that the model fitting was not perfect. Hence, in addition to the chosen model, we also evaluated Gompertzian and exponential growth models, which delivered the same results with regard to the primary endpoint.

Among the interesting questions arising from this study is whether the growth of recurrences could be promoted by contralateral cancer treatment.

First of all, there was no apparent difference in the patient or tumour characteristics between patients with contralateral recurrence vs ipsilateral recurrence or primary cancer, although the small sample size minimizes the certainty of this observation.

Despite our hypothesis that recurrences would grow faster, no significant differences were noted in the growth rate of primary cancers and ipsilateral recurrences. This is consistent with the results of previous studies that have provided solid evidence that radiation therapy decreases the risk of locoregional recurrence, and is associated with improved survival in high-risk patients with breast cancer [18]. Moreover, women who have received chemotherapy or hormonal therapy do not seem to have higher tumour growth rates than women who did not receive such therapies. Cancer treatment at curative dose does not seem to accelerate late recurrence growth in treated breasts [19].

Finally, radiation therapy is the only factor that differentiates contralateral breasts from breasts treated for primary cancer. Although previous studies suggested that radiation therapy can promote accelerated repopulation[20] and radiation-induced breast cancer, particularly in young women, data regarding the radiation therapy associated risk of contralateral breast cancer are controversial [21-24].

A review of randomized trials reported an excess of cancer incidence among women allocated radiotherapy that mainly involved contralateral breast, during a 5-14 year period after randomisation, including among women aged 50 years or older [18].

Several hypotheses can be put forward to explain the potential harmful effects of radiation therapy on contralateral breast cancer.

During external beam therapy of malignant breasts, the contralateral breast receives radiation due to leakage from collimator and scatter from primary. The dose to the contralateral breast has been estimated to be around 5 Gy for 50 Gy primary breast dose [25-27].

Ionizing radiation on contralateral breasts results in DNA and stromal injuries that can accelerate tumour growth [1,28].

Another possible explanation for increased cancer growth may be systemic effects of local radiation therapy mediated by immune or inflammatory systems such as TNFα [29-31]. Indeed, long-term production of TNFα at infraclinical levels is capable of promoting carcinogenesis.

## Conclusion

Despite its retrospective uncontrolled design and small sample size, this study suggests that late contralateral breast cancer recurrences may have accelerated growth. This may be provoked by prior radiation therapy in the primary treated breast. Consequently, newer radiation therapy techniques should be investigated with regard to their roles as risk factors for secondary breast cancer in the contralateral breast.

## Competing interests

The authors declare that they have no competing interests.

## Authors' contributions

IM, DH, EP, DA, and PT were involved in the conception and design of the study. EP and DH enrolled patients in the study and helped to draft the manuscript. IM and DH supervised the study and were in charge of the statistical design of the study. DH, EP, and IM were involved in the provision of patients and data acquisition. IM, DH, DA, and PT were involved in data analysis and interpretation. DH, EP, IM, DA, and PT were involved in writing the report. All authors approved the final version.

## Pre-publication history

The pre-publication history for this paper can be accessed here:

http://www.biomedcentral.com/1471-2407/11/155/prepub
